# Automatic High-Bandwidth Calibration and Reconstruction of Arbitrarily Sampled Parallel MRI

**DOI:** 10.1371/journal.pone.0098937

**Published:** 2014-06-10

**Authors:** Jan Aelterman, Maarten Naeyaert, Shandra Gutierrez, Hiep Luong, Bart Goossens, Aleksandra Pižurica, Wilfried Philips

**Affiliations:** 1 IPI-TELIN-IMINDS, Ghent University, Ghent, Belgium; 2 BIO-IMAGING Lab, University of Antwerp, Antwerp, Belgium; 3 MEDISIP-ELIS-IMINDS, Ghent University, Ghent, Belgium; University of California San Francisco, United States of America

## Abstract

Today, many MRI reconstruction techniques exist for undersampled MRI data. Regularization-based techniques inspired by compressed sensing allow for the reconstruction of undersampled data that would lead to an ill-posed reconstruction problem. Parallel imaging enables the reconstruction of MRI images from undersampled multi-coil data that leads to a well-posed reconstruction problem. Autocalibrating pMRI techniques encompass pMRI techniques where no explicit knowledge of the coil sensivities is required. A first purpose of this paper is to derive a novel autocalibration approach for pMRI that allows for the estimation and use of smooth, but high-bandwidth coil profiles instead of a compactly supported kernel. These high-bandwidth models adhere more accurately to the physics of an antenna system. The second purpose of this paper is to demonstrate the feasibility of a parameter-free reconstruction algorithm that combines autocalibrating pMRI and compressed sensing. Therefore, we present several techniques for automatic parameter estimation in MRI reconstruction. Experiments show that a higher reconstruction accuracy can be had using high-bandwidth coil models and that the automatic parameter choices yield an acceptable result.

## Introduction

In this paper, a novel MRI reconstruction algorithm is presented. The current state of the art in MRI reconstruction consists of many excellent algorithms, but these algorithms require manual intervention for one or more parameter settings, which can be a significant downside. Parameters encompass things such as denoising vs datafit strength, calibration region selection, restrictive k-space trajectory input, pMRI autocalibration kernel size, etc. They arise because reconstruction algorithms attempt to tackle important problems that are associated with different types of MRI acquisition and reconstruction. Many different algorithms exist to cope with different MRI reconstruction problems, but few approaches exist that attempt to jointly solve as many MRI reconstruction problems as possible, fewer still exist that can do this without user parameter tuning.

### Automatic MRI reconstruction

The first goal of this paper is to present a single reconstruction technique that tackles a very wide scope of typical reconstruction problems jointly and automatically: Problems associated with advanced MRI reconstruction are sub-Nyquist sampling (Section 1.1), non-uniform sampling (Section 1.2), noise (Section 1.3) and (autocalibrating) parallel imaging (Section 1.4). The current solutions to these problems entail respectively compressed sensing reconstruction (including but not limited to [1], [2]), regridded reconstruction (including but not limited to [2], [3]), (image-domain) noise estimation [4] and different pMRI techniques [5].

### A new autocalibration formulation

The second goal of this paper is to present a new autocalibrating pMRI formulation. Current autocalibrating pMRI techniques [5]–[9] focus on finding a calibration kernel, which is necessarily of a very limited support. This necessity arises from the need to solve a well-determined linear system to obtain the calibration kernel. We will demonstrate how the large support kernels are a better model for the physics of an MRI acquisition coil system than limited support kernels, which has a detrimental impact on image reconstruction quality. We will also demonstrate how calibration kernels in many current pMRI techniques correspond to ratios of coil sensitivity profiles (or even ratios between coil sensitivity profiles) in the spatial domain, which makes a limited support approximation (or a smoothness assumption) even less accurate. Another class of existing pMRI autocalibration techniques such as [10], [11] focuses on solving the problem of joint estimation of both image and coil sensitivity profiles in image domain. However, this leads to an inherent non-convex optimization problem, i.e. no guarantees can be given on the quality or optimality of the result.

In contrast, we developed a new formulation, that allows for the estimation of calibration kernels with a larger support, because of the inclusion of a regularization term. We directly relate these to the final image, in order to avoid the problem of having to enlarge the allowed support because of the need to consider ratios of coil profiles. On top of that, our new formulation leads to a convex optimization problem, that yields best effort results consistently, without user intervention. We will show that this results in improved autocalibration and as a result, better image quality.

### Paper organization

In short, we aim to design an MRI reconstruction algorithm that is able to cope with pMRI data, that was acquired on a non-uniform grid in an arbitrary trajectory, in a sub-Nyquist way, which can do autocalibration and which automatically tunes all required parameters without user intervention.

This paper is organized as follows: in Section 1, we discuss the aforementioned hurdles in MRI reconstruction, the solution to which will be the focus of this paper. In Section 2, we discuss previous work and existing MRI reconstruction techniques. The proposed MRI reconstruction technique is detailed in Section 3, which consists of an image reconstruction technique (Section 3.1), a pMRI autocalibration technique (Section 3.2) and various techniques to estimate parameters such as autocalibration area, noise level and even field of view (Section 3.3). We demonstrate the proposed algorithm in Section 3.3.

## Methods

### 1 Difficulties in MRI reconstruction

**Figure 1:**
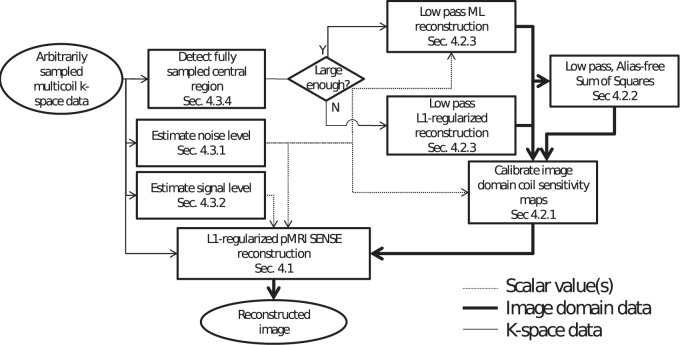
Block schematic of the proposed autocalibrating MRI reconstruction technique.

In this section, we highlight the different challenges and current limitations in the field of MRI reconstruction. We will then propose a solution to these problems in a unified method in Section 3. We solve the Nyquist limit problem by using a regularized MRI reconstruction akin to the compressed sensing MRI literature, described in Section 3.1 with a non-uniform Fourier transform operator, to account for possible Non-uniform sampling patterns. We solve the problem of noise by doing both an estimation of the noise level, described in Section 3.3, and an automatic regularization with a strength based on this estimate, described in Section 3.1. Finally, we solve the problem of calibrating parallel imaging, by supporting both explicit knowledge of coil profiles (as in SENSE) and by proposing a novel autocalibration technique in case this knowledge is missing in Section 3.2. The flowchart of this method is shown in Figure 1.

#### 1.1 The Nyquist limit

The Nyquist limit is a cornerstone concept in sampling theory. It governs the requirement for any continuous signal to be representable in a discrete space without ambiguity. This makes it vital for digital processing of continuous (acoustic, electric, etc.) signals. In MRI, a radio-frequency (RF) electrical signal is sampled and further processed by a digital reconstruction algorithm. For such a reconstruction to unambiguously represents the continuous signal, there are two basic requirements. The signal field of view (FOV) must be finite in length (i.e. periodic repetitions of a finite-length signal), as computers do not have infinite memory. The equivalent result in terms of Fourier space (k-space) is that it suffices to sample this Fourier space in discrete steps, this is the famous Fourier series. Because of symmetry in Fourier transform formulas it is then conversely required that the bandwidth of a signal (i.e. the ‘length’ in Fourier space) is limited, so as to enable a sampling of image space in discrete steps. As such, there are two limitations on MRI sampling. Expressed in terms of k-space sampling, these are:

1. A length limitation (i.e. the MRI image is a 2D/3D signal with finite signal bandwidth).
2. A discretization step limitation (i.e. the assumption of the MRI image as representing an object that fits in a finite FOV).

These are called the Nyquist limits. If the discretization step is too large, the aliasing artifact appears inside the FOV. If the length (the width) of the k-space is too small, the image will appear blurry.

#### 1.2 Non-uniform sampling

As long as a k-space sampling discretization follows the Nyquist limits, the reconstruction constitutes a well-posed problem. In order to simplify reconstruction, a Cartesian sampling is often used. Even if the discretization of this Cartesian grid is non-uniform, useable images could be reconstructed by reweighing the coefficients in a 2D DFT algorithm. However, Cartesian sampling limits the inherent flexibility (and with it the achievable quality) of an MRI scanner, as Cartesian trajectories are only a subset of the space of possible trajectories. It is well known that the best performing trajectories do not belong to the subset of Cartesian trajectories [12], [13]. So, in order to be able to reconstruct the full range of possible k-space trajectories, a reconstruction algorithm needs to be able to deal with non-uniform sampling.

#### 1.3 Noise

Noise in MRI arises mainly from thermal noise in the quadrature RF receiver of the system. As such, it has been accurately modeled by a complex Gaussian distribution on the k-space measurements [14]. The dynamic range of a k-space MRI signal is heavily dependent of the type of acquisition, so is the signal-to-noise ratio (SNR). Because of the large variety of SNR in practical MRI, automatic noise estimation algorithms are desired, while most algorithms still rely on time-consuming and labor-intensive manual tuning of a regularization parameter, which is not particularly optimal and biased towards a particular user.

#### 1.4 Parallel imaging calibration

In MRI, the acquisition time is mainly governed by the repetition time (TR). For each TR cycle, a path in k-space is measured. Therefore, if less points in k-space are measured, then less cycles are needed, so the acquisition time is shorter. In parallel MR imaging (pMRI), an insufficiently densely sampled k-space is deliberately acquired, in order to shorten acquisition time. As explained in Section 1.1 this violates the Nyquist limit and would normally cause the aliasing artifact. However, in pMRI reconstruction data is combined from multiple receiver antenna coils (instead of one) in order to reconstruct an aliasing-free image. Algebraically, the ill-posedness that is responsible for the ambiguity, i.e. the aliasing, is avoided due to the addition of multiple linearly independent data acquisitions. Solving such a system of linear equations requires knowledge about the exact nature of the system, therefore it is necessary to know the sensitivity profile of the receiver antenna coils in the system. There are two basic ways to achieve this goal: first is by actually measuring the profiles of the different receiver antenna coils (SENSE type), second is by extracting a rough estimate from the available data, using a small calibration area in the data (autocalibration, GRAPPA type). An automatic algorithm should be able to deal with both techniques.

### 2 Previous work

Since the early 90 s, the goal of accelerated MRI acquisition has led to the development of different pMRI methods [5]. These include SENSE [15], [16] and variants, the most modern of which bring this line of research into the realm of compressed sensing [17], [18]. Other methods [7]–[9], [19] attempt the harder problem of autocalibrating MRI, in methods that can be better described as marrying GRAPPA [5] to CS MRI [1]. These have the advantage of not needing explicit knowledge of the coil sensitivity profiles as SENSE-based techniques do. They solve this by extracting knowledge of so-called interpolation or calibration kernels from a fixed region of k-space known as a calibration region. Still other techniques, try to marry autocalibration to SENSE and CS MRI, by introducing a joint optimization of both the image and the coil sensitivity profiles in image domain [10], [11].

#### 2.1 K-space (auto)calibration and deconvolution

The calibration problem is severely ill-posed, multiple image-sized coil sensitivity profiles need to be estimated from one single, incomplete number of measurements in k-space. The only way to solve this is to regularize this sufficiently, so as to turn the problem into a well-posed problem. The main drawback of calibrating a k-space kernel is the lack of an efficient way to encode prior knowledge about images, we know a lot about images (with models such as [20]–[25]), but less about what k-space should look like. The result is that [5], [7]–[9], [19] resort to impose prior knowledge in a hard way, i.e. by fixing the bandwidth, i.e. the kernel size, of a k-space interpolation kernel to a very small size. This conditions the problem such that a solution can be reached when sufficient calibration data is available. In classic k-space autocalibration, an image 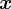 is typically treated as being linked to coil images 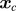 by the equation
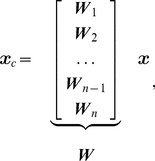
(1)with 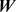 a block diagonal matrix encoding the coil sensitivity profiles. A calibration matrix in a technique such as GRAPPA [6] and SPIRIT [7] or ESPIRIT [9] attempts not at reconstructing the imaged object 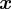, but rather all the images, as seen from the antenna coils 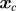, where the final reconstruction is made using a sum of squares. In these techniques, a matrix of the form:
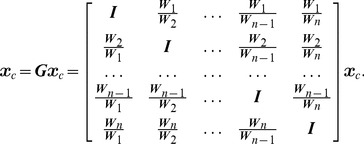
(2)is considered, where we used the notation of the division of matrices to signify element-wise division because the matrices 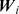 are diagonal and to show that the off-diagonal elements in 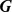 constitute pixelwise divisions. Then, in classic k-space autocalibration, the difficult problem of finding the large number of entries for the diagonal matrices 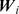 is replaced by the estimation of a small kernel in k-space. The trick is that a (block) Fourier matrix 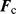 changes the submatrices in 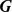 into circulant matrices:
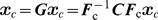
(3)


The assumption in classic k-space autocalibration is that the kernel matrix 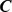 contains many circulant submatrices, where the row(s) of this matrix contain a lot of zero elements, such that the calibration kernels can be considered bandlimited. Once these assumptions are made, the kernel matrix contains 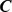 so few independent entries, that these can be estimated using a predefined set of calibration data. Such a matrix 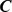 can be used to condition the pMRI reconstruction problem in such a way as to yield good (in the sense of calibration-consistency) image reconstruction. This calibration of 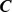 and how 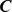 is used to find 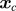 is the difference between the different conventional k-space autocalibration techniques [6], [7], [9]. The difference with the proposed method, is that we are estimating the coil profiles in 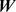 directly, without imposing bandwidth restrictions. The next section explains why.

#### 2.2 Limits to classical k-space autocalibration

The quantities 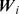 in (1) and (2) are actual spatial sensitivities of a given receiver antenna coil with index 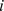 to a pixel (voxel) position. These adhere to Maxwell laws of electromagnetics, but are not trivially found because of the near-field nature of the coils in an MRI system with respect to the object in the scanner. However, we can say a few things about them: they are generally smooth, because the coil size is limited in terms of electrical length. This smoothness is the reason why the bandlimited approximation of profiles from classic k-space autocalibration (Section 2.1) works in the first place.

**Figure 2:**
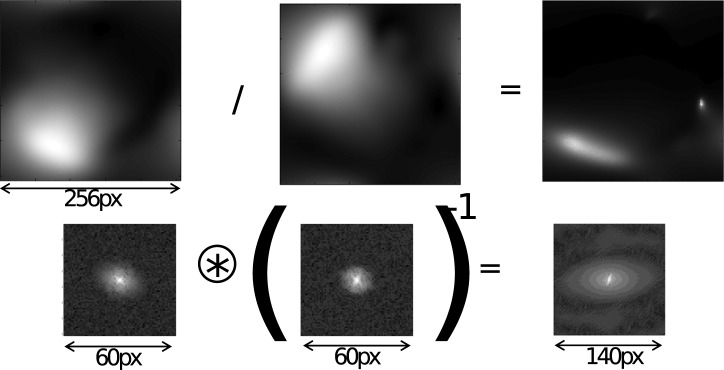
Bandwidth of a spatial sensitivity profiles and calibration kernels. Top row: two coils profiles and their (regularized) pointwise division. Bottom row: corresponding operations in Fourier/k-space. The images were taken from a SENSE dataset. Note how the Fourier domain support size of the division is both larger than that of the typical calibration kernel and larger than the support size of the Fourier domain support of both coil profiles.

However, even if it would be possible to model coils as the simplest antenna possible, i.e. a dipole antenna, the sensitivity profile would decay radially in a 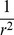 fashion, with 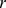 the radial distance from the antenna center. Even this simple 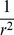 sensitivity profile can not be described as a bandlimited signal (the Fourier transform of a 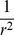 signal is actually a slow linearly decaying function in terms of frequency), it cannot be accurately described using a 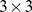 or 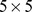 or indeed any kernel of compact support. Instead, we propose to model these profiles as smooth, but not strictly band-limited. On top of that, note how the formulation of 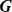 involves pixelwise division of these profiles, which is related to a convolution in Fourier space, enlarging the bandwidth again. We show an illustration of these principles in Figure 2. The top row shows two coil profile estimates (magnitude is shown here) that were taken from a SENSE dataset, and their pointwise division was made, to emulate the behavior of (2). The bottom row shows the cropped k-space magnitude of these images, shown on a logarithmic intensity scale. Although they are indeed smooth, and can be cropped significantly, it can be seen that it is inaccurate to crop these to a very small value such as 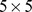 or 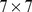. In Section 3.2, we attempt to give a different solution to this problem of autocalibrating MRI with the aim of increasing kernel support size (or rather bandwidth of the spatial domain sensitivity profiles) significantly and with it accuracy.

#### 2.3 Image domain autocalibration

In [10], the coil sensitivity profiles are parametrized to polynomials of low degree. We propose to view this as an implementation of the “smooth, but not bandlimited” model for calibration kernels that we proposed in Section 2.2. However, in this method, there is no distinction between autocalibration data and subsampled data, and the reconstruction is simply formulated as:
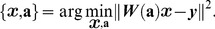
(4)with 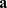 the small set of polynomial coefficients used to model the coil sensitivity matrix 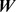 and 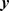 the vector listing the k-space measurements (the input data). Note that (4) is a highly non-convex optimization problem. Therefore, no guarantees can be given on the convergence to a global optimum. A constraint, to force the solution to a desirable one, is made by tuning the allowed degree of the polynomials used for modeling. However, non-Cartesian MRI can vary greatly in subsampling percentage, sampling density variations and noise levels (see Section 1), and since the method does not automatically find an autocalibration region or tune to noise level estimation, aliasing will always interfere with the estimation and the tuning becomes non-trivial and will have to be done by hand. Our proposed method attempts to deal with both problems.

### 3 Proposed technique

An outline for the proposed technique is shown in Figure 1.We will now proceed with a breakdown of this algorithm in a block per block fashion. We will assume knowledge of just the k-space coordinates 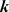, and their corresponding data 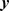.

#### 3.1 pMRI image reconstruction formulation

For the image reconstruction process, we will adopt a regularized SENSE-like approach [16]–[18]. We use this terminology to emphasize that we first want to find image-domain representations of our coil sensitivity profiles and then reconstruct an image with this explicit knowledge. The regularization in this approach is reminiscent of compressive sensing MRI [1], [2], [7], [17]. It is formulated as an optimization problem:
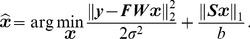
(5)with 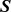 some sparsifying image transformation, or more correctly the basis vectors in 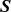 constitute a frame (i.e. it can be a “collection” of multiple image transformations that can jointly make one Parseval frame) that allows to concentrate image energy such that the resulting coefficients are uncorrelated and marginal statistics are well approximated by a Laplacian distribution. This allows for powerful regularization of natural images [26], [27]. 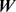 is a block matrix that consists of diagonal matrices to model the pixel-wise multiplication with a coil sensitivity profile. If the coil sensitivity profiles are known (i.e. it is a SENSE experiment) the algorithm simply entails the solution to (5), if they are not known, autocalibration is needed which is the topic of Section 3.2. 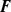 is a block diagonal matrix of non-uniform Fourier transforms (NUFT) [28] that transforms each coil image into k-space data corresponding to measurements from that coil, as such they encode the k-space coordinates 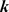. The use of the NUFT is motivated by the desire to also support non-uniformly sampled k-space acquisitions, Section 1.2.

Instead of using a single regularization constant, which is the common practice, we chose a formulation with both a noise level 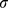 and 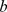. The implication is that (5) is a Maximum a Posteriori (MAP) estimator as a log-likelihood maximization of a Gaussian distribution on the measurement data, which is a known accurate model for MRI noise, and Laplacian prior distribution on the individual transform coefficients, which is known to be very effective in image restoration. The reason we chose this interpretation is that now, we have an idea about estimating the regularization parameter, which is the goal in Section 3.3.

#### 3.2 Autocalibration formulation

In contrast to most autocalibration techniques (see Section 2.1), we do not propose to calibrate a kernel on k-space. In short, strictly bandlimited models are not desirable as a model for calibration kernels because they impose prior knowledge of smoothness in a hard way.

**Estimating the coil sensitivity profiles:** Instead of the standard approach of compact (strictly bandlimited) calibration kernels, we propose to estimate the image space multiplication maps 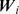, in relation to the original image 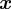, and at a high(er) resolution. We thereby avoid the problem of non-convexity using regularization. This enforces smoothness in a soft way, rather than using the hard, strict bandlimited approximation of existing techniques. The resulting calibration formulation is:
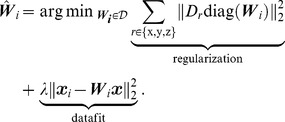
(6)with 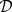 the space of diagonal matrices and 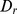 the finite differences operator in 3 dimensions. This introduces a parameter 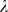, which we chose via continuation in an L-curve technique [29]. This is possible because the estimate of the noise level in Section 3.3 allows to estimate the expected error in the datafit term of (6).

**Finding a preliminary estimate for the image:** The remaining problem is the estimation of 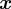 and 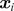, which results in a chicken-and-egg conundrum, because in order to reach this goal, the weights 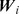 are needed in the first place. The difficulty is two-fold, firstly the overall scaling of the image 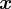 in contrast to the coil images 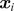 is unknown. There is no real solution to this problem, so we propose the same assumption as in other pMRI reconstruction techniques [6], [7], namely that reconstruction is made through the sum of squares (sos) of the coil images so that for a pixel with index 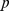, we sum over the coils 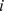:
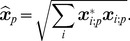
(7)


The implicit assumption is that the sum of squares of the coil sensitivity profiles 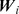 is constant. The advantage of the assumption (7) is that is optimal with respect to maximizing SNR of the end result [30]. Secondly, in the impossibility of a joint (pMRI) reconstruction, any reconstruction of 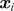 (and as a result 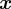) will be corrupted by the spatial aliasing artifact.

**Finding a preliminary estimate for the coil images:** The remaining problem is to find an estimate for the images 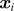. We cannot estimate these by straightforward LS-NUFT ([3]) reconstruction, as this would lead to spatial aliasing because of the possible sub-Nyquist sampling of the k-space. We propose to avoid the spatial aliasing artifact by only reconstructing the part of the image k-space that is fully sampled. In a way, this is an automatic method for detecting data on which calibration is possible. The details of this technique are given in Section 3.3. The coil images 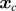 are obtained through least-squares estimation from the k-space data using:
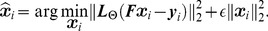
(8)


with 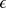 a small constant to avoid ill-posedness and 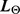 the operator that isolates the set of k-space data points 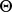 that make up a fully sampled region as given by the technique in Section 3.3. Finally, in the unlikely case that there are insufficient points in 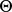 to obtain aliasing-free coil images with reasonable resolution, we resort to a failsafe “L1 regularized” reconstruction mode, which uses a classic CS formulation [2] to avoid spatial aliasing:
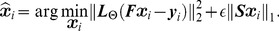
(9)


Note that this is similar in goal to the technique proposed in [31], where Thikonov regularization was used regularizing wavelet coefficients, while we use L1 regularization on shearlet coefficients. The choice for approximate perfect data-fit here, is motivated by the high SNR nature of these low frequency k-space points. The decision on using (9) instead of (8) is made when the estimated FOV/resolution from the method in Section 3.3 is smaller than the user-defined FOV/resolution.

#### 3.3 Parameter estimation

Ideally, we would recommend to optimize the parameters in (5), in order to minimize the mean squared error with the ground truth. In the absence of ground truth (i.e. in a realistic application), an excellent alternative is to minimize the Stein unbiased risk estimate (SURE) [32]. However, this require iterative evaluation of the reconstruction algorithm in order to properly estimate the SURE, which can be time-consuming. In this work, we seek to optimize the result for a single run of the reconstruction algorithm, note that this output could be used as an initialization for a SURE-based parameter estimation algorithm. We seek to do this through interpretation of the parameters 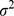 and 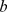 in (5) as respectively ‘noise level’ and ‘signal level’.

**Figure 3:**
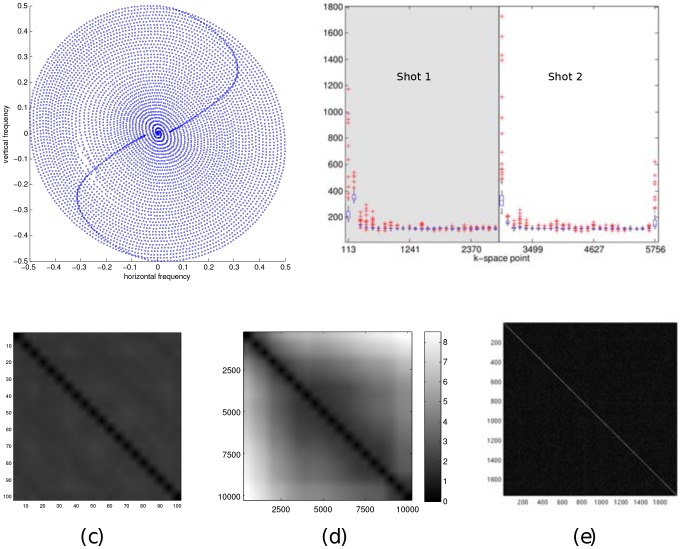
Results from noise measurements on 10 000 repeated acquisitions of a 2 shot spiral: the trajectory (a) box plot of per 100 measurement variance estimates on each k-space point (b) the absolute error matrix between two sets of 100 measurements over a short timescale (c) the absolute error matrix between two sets of 100 measurements on a longer timescale (d) the crosscorrelation matrix between k-space points overall (e).

**Figure 4:**
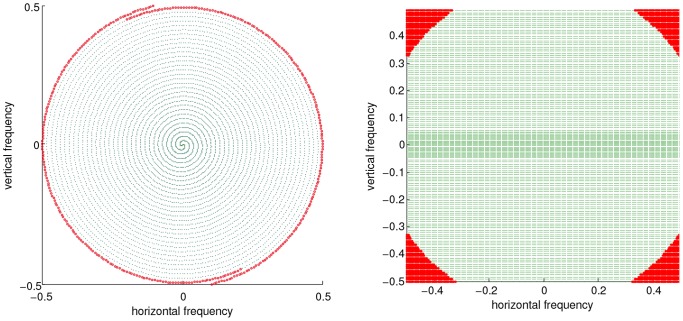
K-space points considered for noise level estimation indicated in red. Left: Subsampled spiral, Right: pMRI GRAPPA sequence.

**Noise level estimation:** The ‘noise level’ or rather the parameter as defined in our estimator (5) as 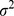, is a measure for the noise variance in the k-space data. MRI noise can be considered complex Gaussian [14]. Commonly, noise samples are also treated as being uncorrelated. We've put the whiteness assumption to the test. The autocorrelation matrices of a repetition experiment is shown in Figure 3. We can draw several conclusions from this, the first is that the noise variance is quite stable as k-space is traversed. Nonetheless, there seems to be an increase in the estimated noise variance for the points in k-space closest to the center (seen in Figure 3(b)), we attribute this to an inconsequentially small non-linearity in the receiver system, because of the huge dynamic range of low-frequency k-space points. Another conclusion is that the noise within one acquisition sequence can safely be assumed to white, this is shown by the correlation matrix between samples within one acquisition sequence, Figure 3(e). We therefore do not deviate from the common model of white noise. We do pose one caveat here, it seems that the as the timescale of acquisition grows larger, the difference between two acqusitions (the error) becomes larger, we attribute this to heating, compare the short timescale, Figure 3(c) with the large timescale, Figure 3(d). Therefore, it is prudent to estimate the noise for each acquired dataset, which we will do in this work. However, the presence of a signal component to the data will still hinder accurate noise estimation. Therefore, we do not simply use the sample variance estimator, we choose a different approach, akin to image denoising literature, where it is a common practice to estimate noise levels in a robust way using the median absolute deviation (MAD) measure [33]. In (multiresolution) image denoising, an image transformed by a sparsifying transform has many coefficients that tend to zero in a noisefree environment, which means that a robust estimator will consider the sparse large coefficients as outliers and a good estimate of the noise variance can be obtained. Similarly, the Fourier transform can be considered as a(n) (somewhat less) effective sparsifying transform, so the k-space data is quite sparse. Again, since the noise model in MRI is white and complex-valued Gaussian on the k-space data, a robust estimator will get us a reasonable estimate of the noise variance 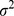. Furthermore, we know that the highest signal energy is concentrated in the center of k-space [34], conversely the lowest signal energy can be found in the periphery of k-space. Therefore in the proposed algorithm, we perform this MAD estimate on the 5% of k-space points that have the highest radial frequency.
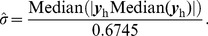
(10)where 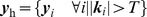 with the threshold 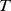 chosen to correspond to the 95% fractile value of the radial values of the k-space coordinates, in other words the 5% of points that are furthest from the k-space center. We show this 5% fraction of k-space points on two sub-Nyquist sampled trajectories in Figure 4.

**Figure 5:**
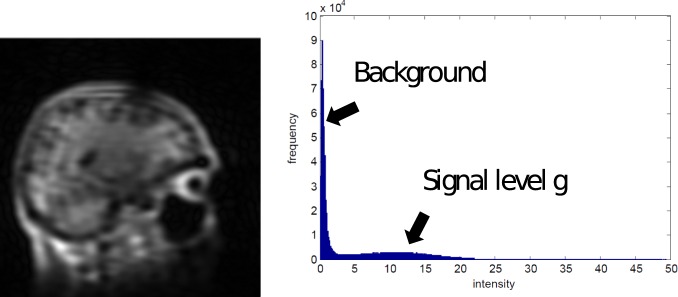
Demonstration of how the signal level 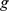 is estimated. A crude reconstruction is made (left), which can contain aliasing, then a histogram is made of its pixel values and a mixture of 2 Gaussian distributions is fit to its histogram (right).

**Signal level estimation:** In order to run the estimator (5), not only the noise variance is needed, which is estimated according to Section 3.3, but also the parameter 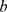. This parameter is interpreted as equivalent to the variance of the Laplacian distribution that is used as a model for the coefficients of the image transform that is applied to the image. If the noise and corruption-free image is known, this can be easily estimated, however in general this is not known. However, it is known that the there is a linear dependency between the 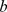 and a hypothetical multiplication factor applied to the image. This is because the image transformation is linear, and the relation between the signal variance 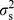 and 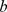 is known to be 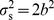. Therefore, any signal that represents an imaged object can be normalized with respect to its average gray value and its associated 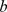 parameter will be rescaled with the same factor. If we consider the broad class of MRI images to have the same level of relative contrast with respect to this average gray value, we can consider this a parameter that is fixed for many different MRI modalities. We propose the following model:
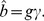
(11)where 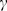 is a signal-independent value that is applicable to all MRI modalities (i.e. the relative signal value, or rather the relative edge strength in a typical MRI image) and 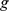 is a signal-dependent average gray level for the MRI image under reconstruction. In order to estimate this 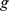 parameter, we look at a preliminary LS-NUFT (sum of squares) reconstruction [3], this will potentially have a significant spatial aliasing artifacts, but as the center of k-space is always fully sampled, these will only consist of high-pass signal components. However, depending on the FOV with respect to the size of the image object, the average gray value is influenced by the large number of background pixels. Therefore, we use an image segmentation technique based on a mixtures of Gaussians model. We fit a double Gaussian model to the histogram of voxel intensity values, where the Gaussian with the lowest mean, will be centered around 0 as it contains the background pixels. The other Gaussian will then have a mean that corresponds to the “signal level” 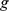 that is of interest here. A demonstration is shown in Figure 5.This approximation allows for a fairly accurate automatic parameter estimate, that can subsequently be tuned manually for extra accuracy.

**Figure 6:**
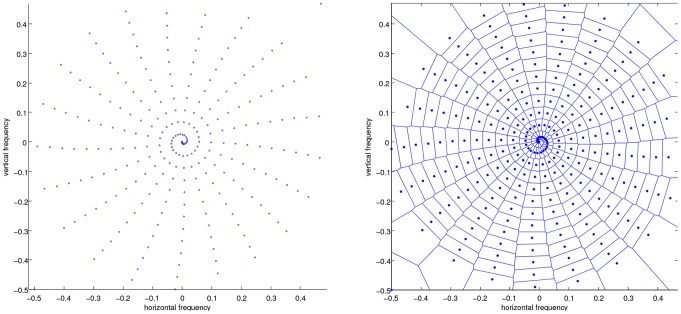
Left, a 2D grid of k-space sampling coordinates, Right, its Voronoi tesselation.

**Field of view estimation:** As k-space sampling density governs aliasing artifact, explained in Section 1.1, the density is the defining factor for the maximum possible field of view without introduction of aliasing artifacts. Of course, in a non-uniform trajectory, this density varies. We propose the following technique, that is reminiscent of some regridding heuristics [35], to estimate a local density measure: Firstly, a Voronoi diagram is made from the grid defined by the k-space coordinates, this process is shown in Figure 6. The complexity for such an algorithm can be 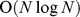, an algorithm called Fortune's algorithm [36]. The center 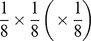 part of k-space is considered to be fully sampled (unless otherwise specified by the user), due to the observation reported by many authors [34], [37], [38] that the lowpass part of an MRI image needs to be fully sampled for acceptable reconstruction to be possible, as it can not be sparsified. So in any useable MRI scan, (at least) this part will be fully sampled. Within this region, the largest Chebychev distance from a k-space point to a Voronoi grid point is considered as the limiting FOV factor for this image (see Section 1.1 for a discussion about the relation between FOV and discretization step). We chose the Chebychev distance 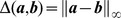 as this is consistent with the way FOV is interpreted on a Cartesian sampled grid: We know that for a Nyquist sampled Cartesian grid, the following holds:
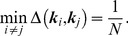
(12)with 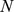 the size of the image in any direction (if the image can be considered as a cube) and the convention is used that the frequency space is scaled such that the Nyquist bandwidth is the unit bandwidth. In a Cartesian grid, the minimum Chebychev distance to a Voronoi grid point is then exactly 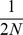. So we propose to use our Voronoi technique to find the largest Chebychev distance to a Voronoi grid point, among the k-space samples inside the center 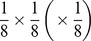 bandwidth (the set of which we call 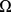) and equate this to 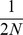. This yields the following estimate for the FOV 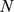:
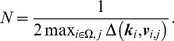
(13)


where 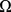 is the set of k-space points that are within the aforementioned region and 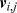 is the 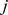-th Voronoi grid point associated with k-space coordinate 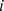. For non-square images, this procedure can be extended trivially.

**Figure 7:**
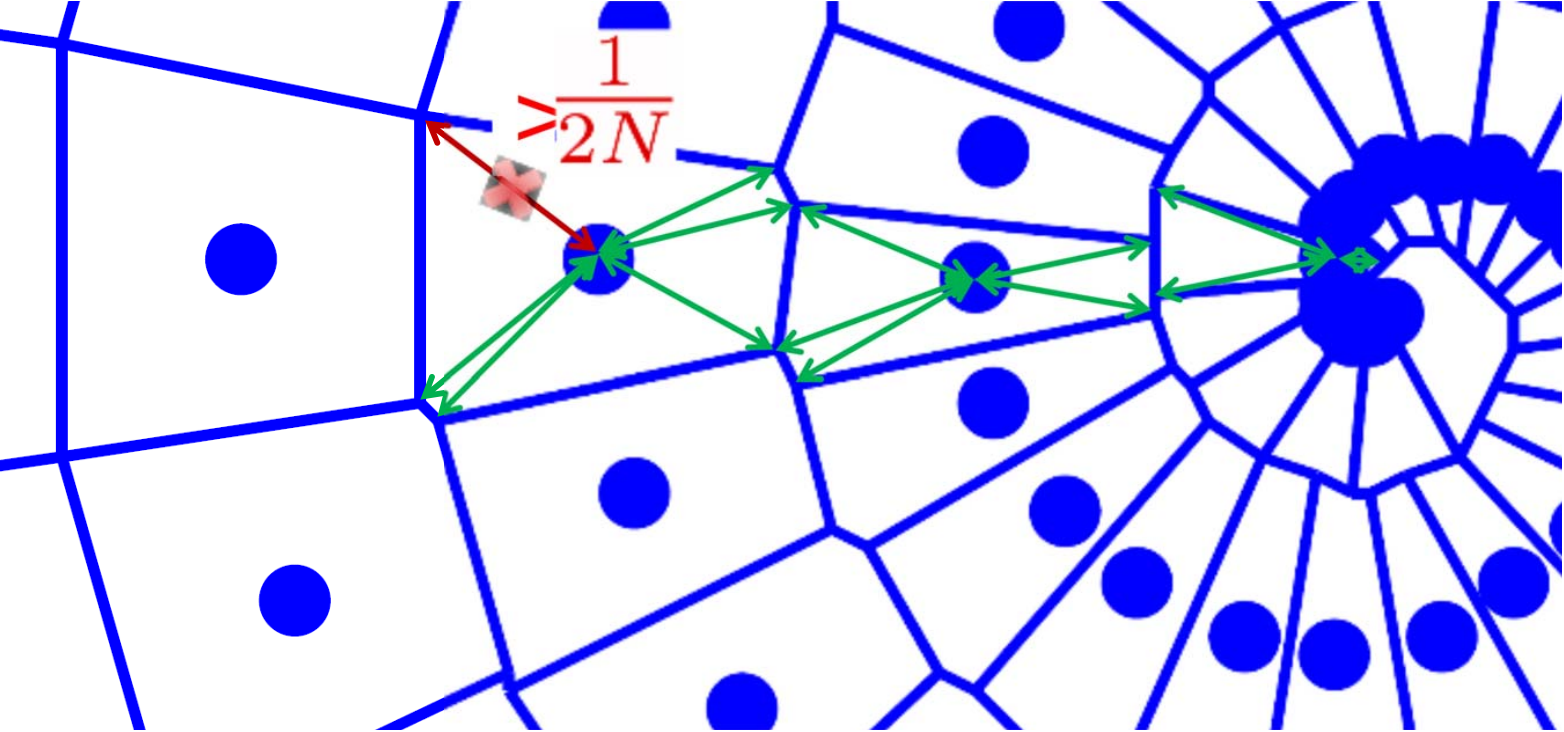
Detail of the Voronoi tesselation in Figure 6 showing the principle of the density estimation.

**Figure 8:**
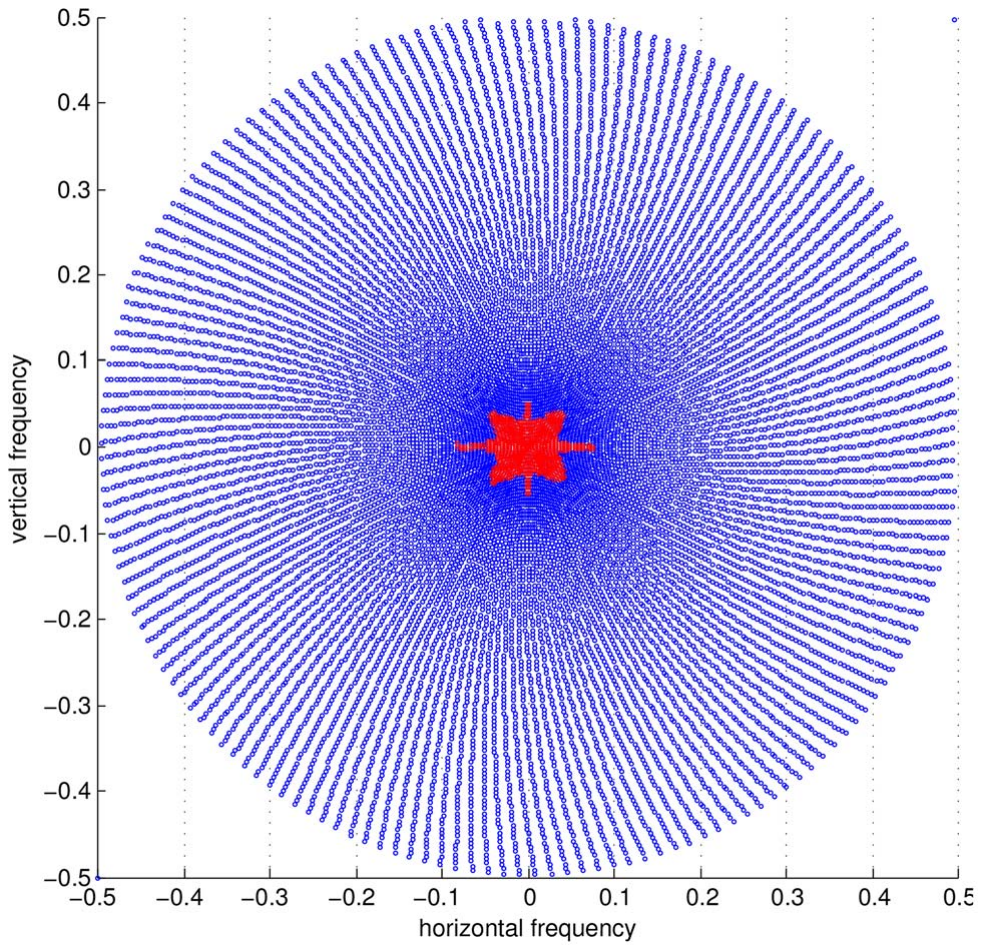
A 25% subsampled Spiral trajectory for a 256×256 image. The blue points are all the data points, the red portion of points signifies the automatically detected autocalibration region.

**Calibration area estimation:** Once the field of view/resolution 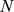 is found using the procedure in Section 3.3 or given by user input, an estimate can be made of the autocalibration area. By autocalibration area, we mean the area (or rather the set of k-space coordinates 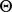) on which autocalibration can be performed by the algorithm described in Section 3.2 in the case of pMRI. The relation between sampling density, field of view and spatial aliasing was described in Section 1.1. As the k-space sampling density fully defines the (Chebychev) distance between k-space points, (13) constrains the allowable FOV. The proposed algorithm for autocalibration requires aliasing-free images, so we will look for the largest area of k-space around the center, that is sufficiently densely sampled to satisfy (13), in order to generate spatial aliasing-free calibration images of the highest signal bandwidth. Again, we propose a Voronoi tesselation based technique to find this area. The procedure is illustrated in Figure 7: The set of autocalibration data is built up in a greedy way, gradually adding points to a whitelist. Starting from the center k-space point 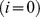 and radially moving out, it is checked whether the following holds:
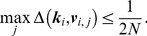
(14)If the condition (14) is violated, then the conflicting Voronoi grid points 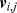 are blacklisted and any k-space points 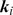 that has a blacklisted Voronoi grid point 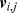 and is not yet on the whitelist will be omitted from consideration into the autocalibrating data set 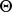, with its remaining voronoi grid points also blacklisted 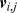. This algorithm is run as long as there are still eligible points to be included into the autocalibration set. The result is a set of k-space points that corresponds to a spatial aliasing-free low-pass approximation of the coil images, ideal to estimate the coil profiles from. An illustration is shown in Figure 8, where a 25% subsampled Archimedean spiral is shown. The distance between two loops of the spiral is 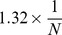. The points in red are the autocalibration set 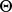 as created by the procedure detailed in this section.

## Experiments and Results

We intend to show two things in this result section: Firstly the increase in accuracy for pMRI reconstruction due to the novel pMRI autocalibration framework, and then secondly, the versatility of our automatic reconstruction algorithm, both with respect to noise robustness and (3D) k-space trajectory. To show the accuracy of pMRI reconstruction, we did several experiments: autocalibrated pMRI in both non-Cartesian and Cartesian setting. To show the effect of noise robustness, we did similar experiments, adding noise to both Cartesian and non-Cartesian acquisitions. Finally, we emphasized the k-space trajectory versatility, by including the aforementioned non-Cartesian acquisition reconstructions, as well as a spiral reconstruction.

### 4 Non-Cartesian autocalibrating pMRI

**Figure 9:**
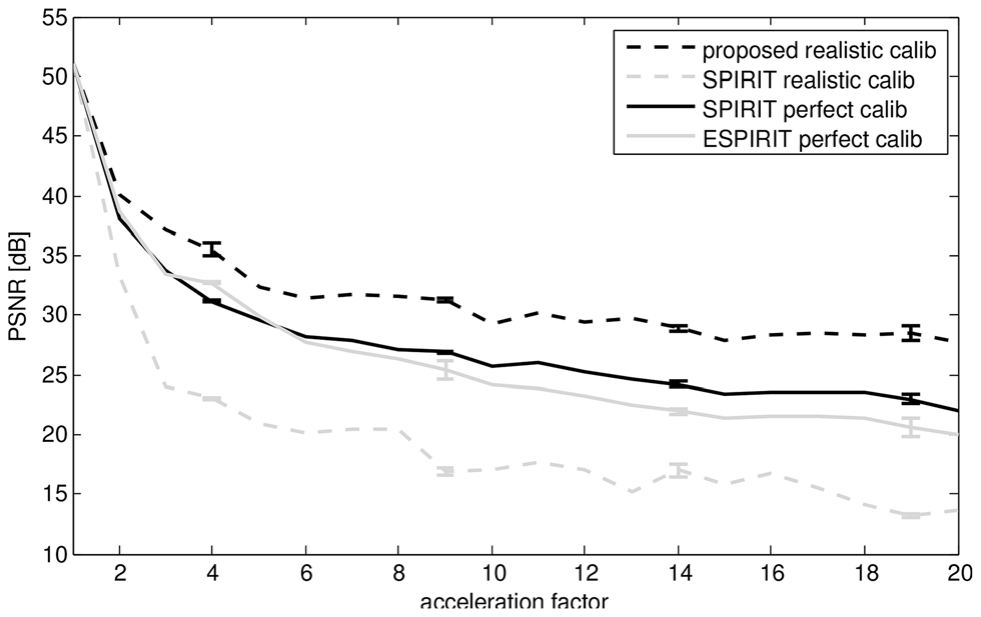
PSNR, in function of acceleration factor, with respect to the reconstruction at acceleration factor 1. The distinction between realistic and perfect calibration is with respect to the data used for calibrating the pMRI reconstruction. Realistic calibration uses only the undersampled dataset, perfect calibration uses the fully sampled dataset, before the simulated undersampling.

**Figure 10:**
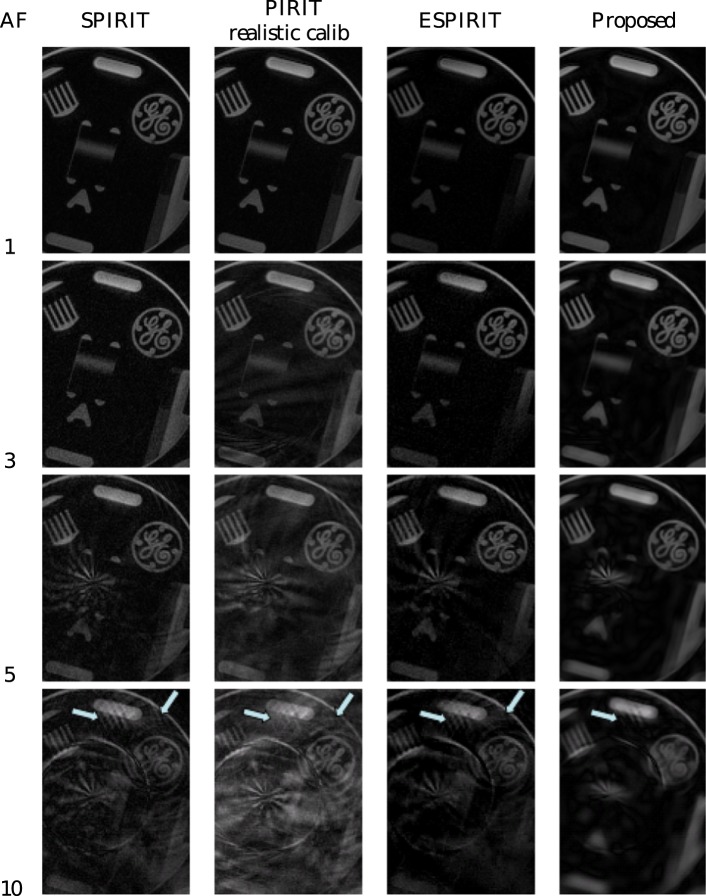
Visual comparison between ‘perfect calibration’ SPIRIT, ‘realistic calibration’ SPIRIT, ESPIRIT and the proposed method for reconstruction of some of the datapoints that make up_ Figure 9 the acceleration factor (AF) is shown on the left.

In a first experiment, we make the comparison to the SPIRIT method from [39] and ESPIRIT from [9], the code of which is publicly available at http://www.eecs.berkeley.edu/~mlustig/Software.html. We replicated the experiment detailed in the code: A spirally acquired hardware phantom, with a fully Cartesian sampled center, is simulated to have been acquired in an accelerated fashion, by decimating the data. The original acquisition was described in [39]: A cardiac antenna coil was used with four channels, the trajectory had 60 interleaves, and 0.75 mm in-plane resolution for a 30 cm FOV. The readout time was at 5 ms, to avoid off-resonance effects. The phantom was scanned on a GE Signa-Excite 1.5-T scanner. The acceleration ( = decimation) factor was varied between 1 (no acceleration) and 20. A comparison of peak signal to noise ratio (PSNR) in function of acceleration factor is shown in Figure 9. PSNR is a quality measure that is equivalent to the mean squared error, it is defined as
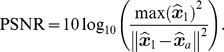
with 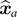 the reconstruction for acceleration factor 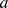, with 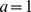 corresponding to the fully sampled reconstruction. We repeated the experiment while shifting the subsampling lattice to check the stability of the reconstruction algorithms. We make a distinction between SPIRIT with “perfect calibration” and SPIRIT with “realistic calibration”. The calibration kernel for the SPIRIT algorithm is calculated on fully sampled data, since our algorithm only uses subsampled data (i.e. with automatic detection of sufficiently densely sampled bandwidth), we made a comparison with a modification of the SPIRIT code that also only uses subsampled data. For this we varied the calibration kernel size and the training data size and kept the best result. This is shown as the “realistic calibration” option in contrast to the original code which we call “perfect calibration”. For a visual comparison of the results from this experiment for different acceleration factors (AF), we refer to Figure 10. This experiment shows that the proposed algorithm suffers from slower and less quality loss as the acceleration factor increases. This can be seen in the spiral artifacts, that are less severe, but also in the spatial aliasing of the comb structure indicated by the arrows, which appears at higher AF and is less severe for the proposed method. Also, the proposed method retains some notion of the dart structure, even at AF 10.

### 5 Noise-robust Non-Cartesian autocalibrating pMRI

**Figure 11:**
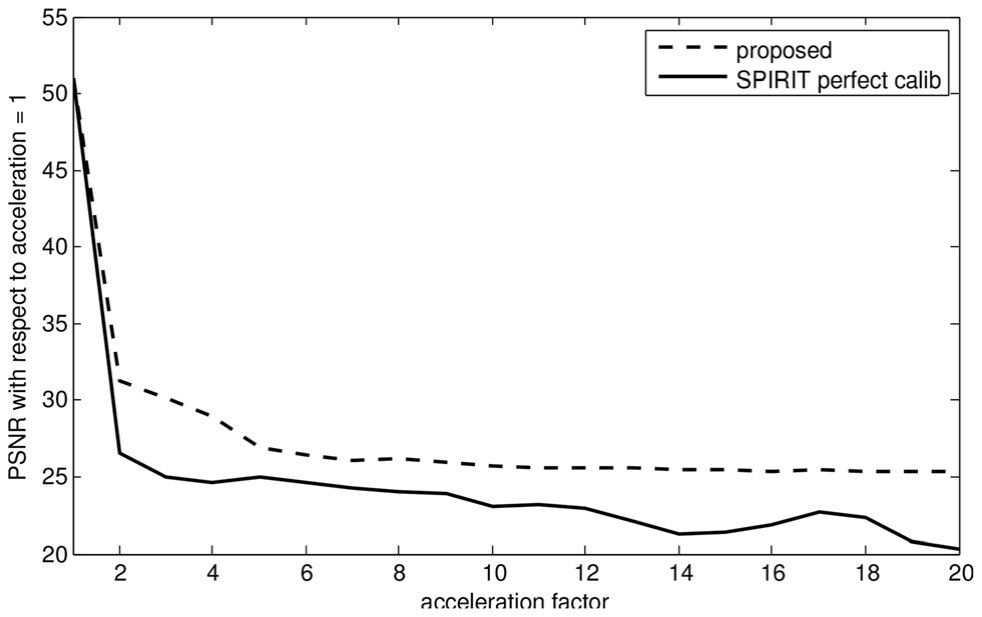
PSNR, in function of acceleration factor, with respect to the reconstruction at acceleration factor 1. The dashed line is the proposed method, the full line is SPIRIT with fully sampled knowledge of calibration data.

**Figure 12:**
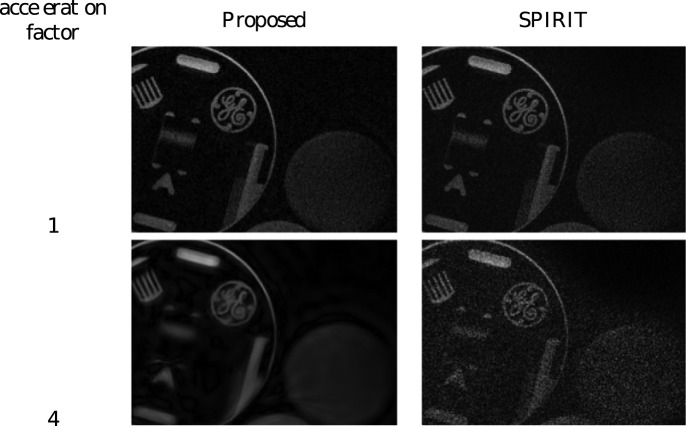
pMRI reconstruction results for the GE hardware phantom: a visual comparison between proposed reconstruction and ‘perfect calibration’ SPIRIT reconstruction for some of the graph points that make up the graph in Figure 11, for AF  = 1 and AF  = 4.

In this experiment, we have repeated the experiment from Section 4, but we added a high amount of white Gaussian noise to the k-space data (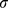 = 40). Since there is no ground truth data with respect to the image that corresponds to this data sequence, we calculated the PSNR, using the reconstruction for each algorithm given a fully sampled dataset (acceleration  = 1) as reference image. The aim is not to show denoising performance, but rather relative degradation in reconstruction performance as the acquisition acceleration increases in a low SNR environment. The PSNR curve in function of AF is shown in Figure 11. Although the proposed algorithm suffers fails to deliver an acceptable reconstruction (we consider ‘acceptable’ as PSNR 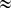
35dB) as the acceleration factor increases beyond 4, it can be seen how the proposed algorithm maintains a high(er) PSNR due to its inherent noise-aware reconstruction. Figure 12 shows two data points from this curve, with AF  = 1 and AF  = 4, where the effect of automatic tuning to the noise level can be seen. It can be seen that the general shapes of the objects is better preserved by the proposed algorithm, where e.g. the outline of the cylinder on the right is not discernible due to noise in the SPIRIT case with AF  = 4. Of course, the algorithm can be tuned to any amount of denoising action, as the denoising parameter can still be adjusted manually.

### 6 Noise-robust Cartesian pMRI

**Figure 13:**
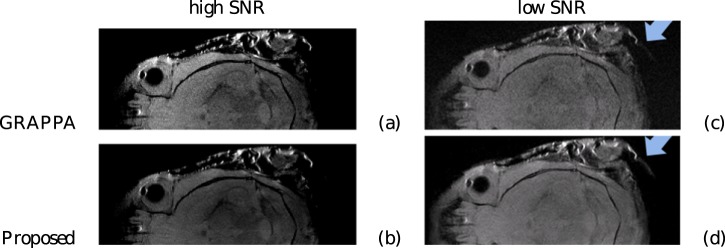
Contrast enhanced reconstruction from GRAPPA experiment. For both the high SNR and the low SNR case the contrast was adjusted for maximal visibility. Top: reference GRAPPA reconstruction, Bottom: Proposed.

**Figure 14:**
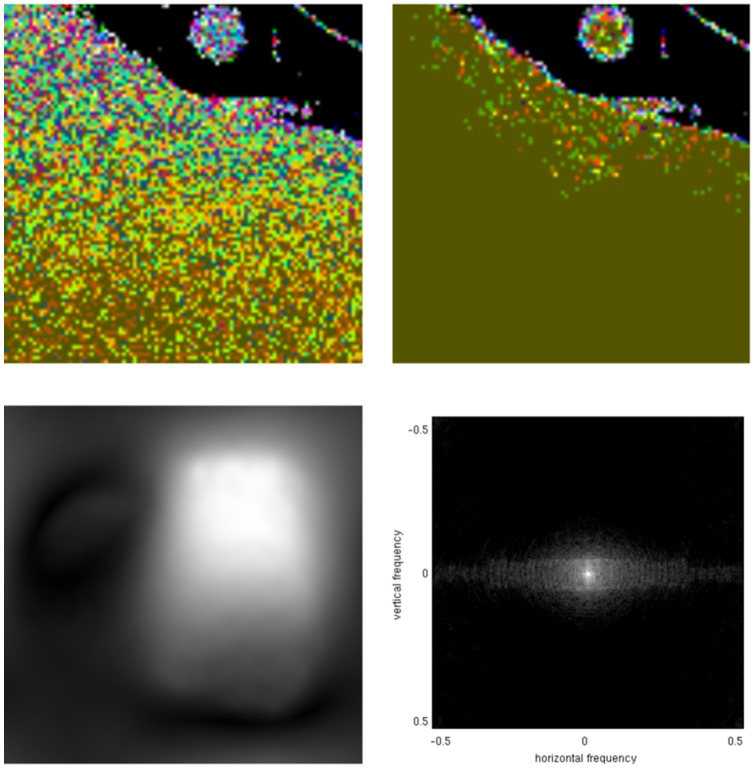
Illustration of an estimated coil sensivity profile. Top left: a crop from the noisy GRAPPA reconstruction, in high contrast indexed colormap Top right: a crop from proposed reconstruction, in high contrast indexed colormap Bottom left: estimated coil profile from coil 1, Bottom right: logarithmically scale power spectral density of the coil profile in the bottom left.

Another experiment is the comparison between the reconstruction from a Bruker GRAPPA acquisition. As usual for GRAPPA, lines were evenly removed from a fully sampled Cartesian grid to result in a sub-Nyquist k-space trajectory that was acquired using 4 receiver coils. The center 10% of the k-space was fully sampled to allow for autocalibration, the remainder was subsampled to 50%. It can be seen in Figure 13 that the reconstruction result from the proposed method is of higher contrast and lower noise level when compared to GRAPPA. This is attributed to the inherent automatic regularization and improved autocalibration model in the proposed method. We should not expect large visual differences here because a pure GRAPPA experiment is well-posed reconstruction, apart from the noise. We then performed an experiment where we added extra noise to the k-space data. Again, the result is shown in Figure 13. It can be seen that the noise robustness is retained, and that some structures, such as the appendage in the top right is clearly visible, while it is barely distinguishable in the reference GRAPPA reconstruction. From this experiment, another interesting observation can be made. We show a crop from this low SNR experiment in Figure 14. It can be seen how the classic GRAPPA reconstruction produces a ‘noise halo’ around the object. We changed the visualization for this to a high-contrast indexed colormap, in order to improve visibility of this halo in print. The halo effect can be attributed to spatial differences in coil sensitivity, but can just as well be explained through incorrect estimation of the coil sensitivity profile, due to the inaccurate small kernel size assumption of classic GRAPPA (Section 2). Note how the proposed method does not produce this ‘noise halo’. We added the estimated coil profile for one of the four coils as an illustration, it is far more complex than could be expressed through limited size kernels, as clearly visible in the power spectral density of this coil profile signal, where the slow spectral decay can clearly be observed, which is more consistent with electromagnetic physics.

### 7 Cartesian subsampled phase encoding k-space autocalibrating pMRI

**Figure 15:**
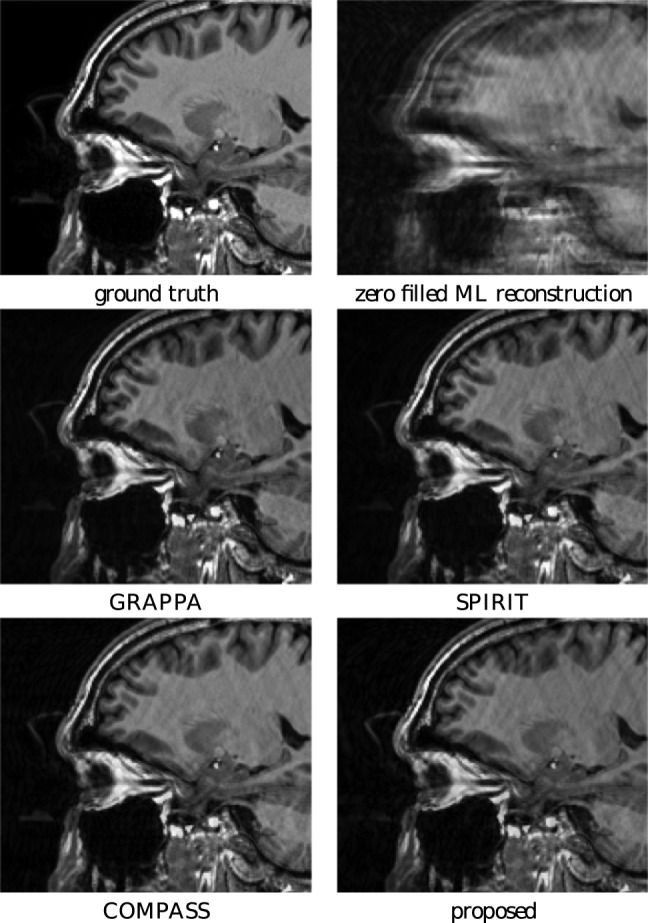
Reconstruction experiment for a simulated random subsampling of phase encoding lines to 34% of the Nyquist rate. GRAPPA, SPIRIT and the proposed method use autocalibration. The COMPASS method uses exact knowledge of the simulated coil profiles.

**Figure 16:**
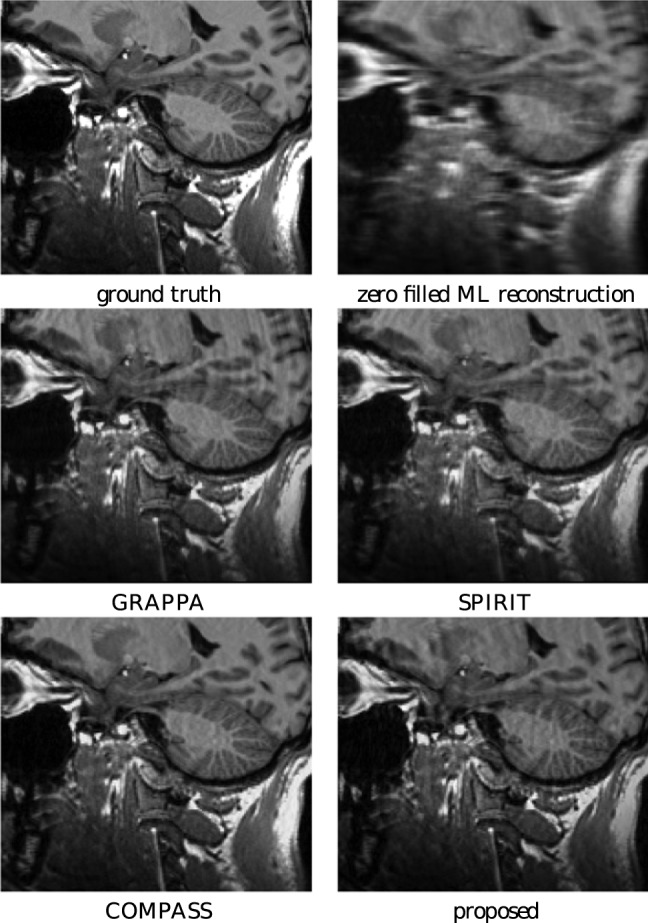
Reconstruction experiment for a simulated random subsampling of phase encoding lines to 20% of the Nyquist rate.

In this section, we perform 2 subsampled simulation experiments for a GRAPPA acquisition: 8 coils were simulated to acquire a software phantom image on a subsampled Cartesian grid. By subsampling the phase encoding direction, the same lines were randomly removed to reduce the dataset to a 34% Nyquist sampled set, where the center 6% was fully sampled. The result of the reconstruction experiments are shown in Figure 15. Next, we did a more difficult reconstruction experiment where the same lines were randomly removed to reduce the set to a 20% sampled Nyquist set, here the center 12% was fully sampled. The result of these reconstruction experiments are shown in Figure 16. Although the reconstruction algorithms start from different assumptions with respect to autocalibration, all techniques yield comparable qualitative results in this experiments, although it should not surprise that spurious artifacts appear in different places due to the different way of autocalibrating. The strength of the proposed algorithm again lies in its versatility, in that the autocalibration area was estimated automatically and that it is applicable beyond the application of MRI reconstruction of Cartesian acquisitions with subsampled phase encoding directions.

### 8 Arbitrarily sampled k-space autocalibrating pMRI

**Figure 17:**
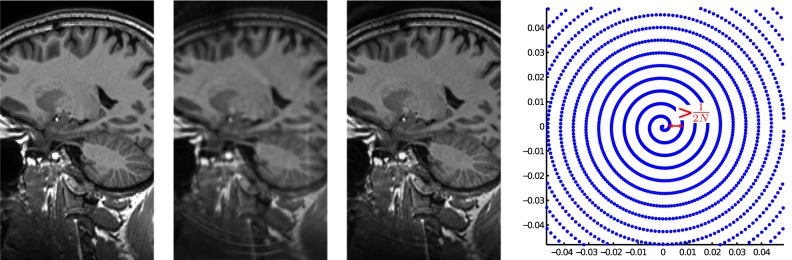
Reconstruction from a simulated subsampled Archimedean spiral pMRI acquisition. (left) Ground truth, (middle left) maximum likelihood sum of squares reconstruction (19.2dB), (middle right) proposed method (21.9dB), (right) k-space sampling pattern detail, notice the spiral arms being too distant to allow for conventional autocalibration.

Another experiment is a simulation on a software phantom of a human head. This phantom was made by taking the central slice from a 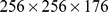 voxel reconstruction from an MPRAGE sequence [40] and simulating non-Cartesian acquisition on it using NUFT software. It was acquired on a simulated Archimedean spiral, with the distance between two loops of the spiral being 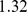 times the Nyquist limit and sampled at even angular spacing such that the total number of samples is 25% the Nyquist rate. The data was simulated as an acquisition using 8 coils with coil profiles taken from a different SENSE experiment. Since in this setup there is no calibration-capable area, i.e. no bandwidth around the origin of k-space where the data is sufficiently densely sampled, the proposed algorithm automatically switches to the l1-regularized calibration algorithm (9). The only reference algorithm we can compare to is a sum of squares from ML reconstructed images. This is because no GRAPPA or SPIRIT kernels can be trained, as a k-space point pattern is never repeated and, strictly spoken, no autocalibration region exists. We can see in the result shown in Figure 17 that even this regularized calibration approach succeeds in avoiding the severe aliasing artifacts that arise with the reference algorithm, while still achieving great contrast and sharpness.

### 9 Arbitrarily sampled 3D k-space autocalibrating pMRI

**Figure 18:**
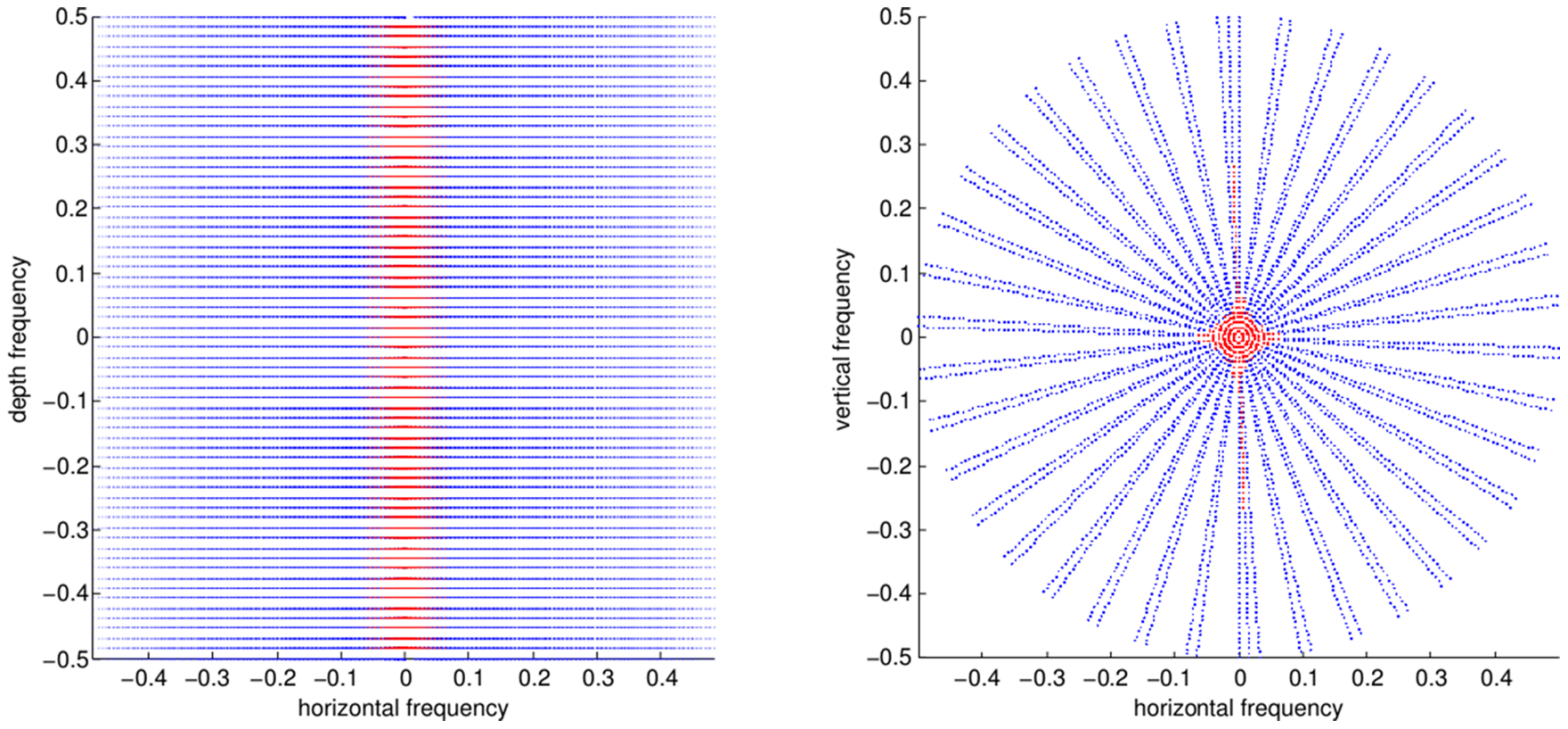
K-space trajectory used in the 3D stack of spiral experiment. The red points constitute the automatically detected autocalibration area. Left: side view, Right: top view.

**Figure 19:**
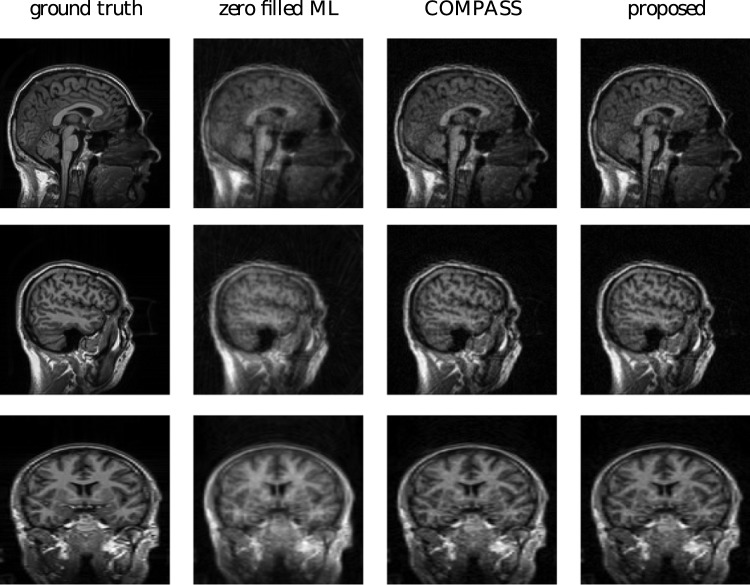
Reconstruction experiments from an autocalibrated 3D stack of spirals reconstruction. Top row: saggital view of slice 32. Middle row: saggital view of slice 16. Bottom row: coronal view of slice 64.

The proposed approach is very versatile and is suitable to work with non-uniform k-space, as we explained in Section 3.1 and demonstrated in experiments. Similarly, the method can be extended trivially to support 3D sampling patterns. As an illustration, we add the result of a 3D autocalibrated pMRI reconstruction experiment. We used the same 3D MPRAGE-based phantom from the previous section. The simulated image acquisition and reconstruction was for a grid of the size 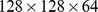 voxels and it was acquired on an irregular stack of radial lines. Two projections of these k-space coordinates, along with depictions of the automatically detected autocalibration region, are shown in Figure 18. The results of the reconstruction experiments are shown in Figure 19. The comparison between COMPASS and the proposed method is interesting because the COMPASS technique [17] is SENSE-like in that it takes complete knowledge of the coil sensitivity profiles as an input. As such, the COMPASS and the proposed method can be used to assess the autocalibration model. The simulation experiment was set up to make this comparison possible, by simulating 8 coils that combine into a uniform profile, when performing a sum of squares reconstruction. The results show that the autocalibration technique succeeds in automatically finding the proper coil profiles, without user intervention, in order to yield a result that is indistinguishable from a reconstruction that starts with full knowledge of the coil profiles.

### 10 Discussion

These experiments have demonstrated that the proposed technique for autocalibration outperforms the existing state-of-the-art techniques with respect to calibration accuracy for non-Cartesian trajectories. This was demonstrated with reconstruction experiments for various acceleration factors in Section 4. It was found that, as the AF increases, the proposed algorithm suffers from slower and less quality loss with respect to the fully sampled reconstruction in comparison to the state of the art. We attribute this to our improved model for and estimation of coil sensitivity profiles, one of which is illustrated in Figure 14.

Furthermore, the proposed technique's image reconstruction was demonstrated to possess (automatically tuned) noise robustness, which was demonstrated both for the non-Cartesian (in Section 5) and the Cartesian (in Section 6) pMRI case. It was also demonstrated how this noise robustness results in significantly better results, in terms of mean squared error as well as some qualitative detail recovery, in comparison to conventional techniques, because these techniques are typically oblivious of noise levels.

Lastly, the versatility of the proposed technique was further illustrated by different experiments, both Cartesian and arbitrarily sampled non-Cartesian, 2D and 3D pMRI reconstruction in Section 7, Section 8 and Section 9. Reconstruction in all this cases is automatic, i.e. without user input. Estimation of calibration area, noise level, calibration kernel, desired FOV or resolution, etc. is all done automatically following the procedures outlined in this paper.

The computation cost for this algorithm is similar to that of other iterative MRI reconstruction algorithms, and it scales linearly with the number of data points. Take the simulation in Section 7 as an example, the total reconstruction time is 170 s for an image of 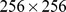. 20 seconds (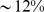) of this is taken up by the autocalibration algorithm and 150 s by the reconstruction algorithm. Note that the time taken up by the autocalibration is low, this is defined by the size of the autocalibration region, which depends on the k-space trajectory and is estimated automatically by the algorithm. In the example, the proposed algorithm detects 3694 points out of 29440 trajectory points (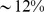) as autocalibration region. This explains the speed of the autocalibration algorithm, as usually less datapoints have to be considered for the autocalibration part. Conversely, if the autocalibration area is found to be large, the autocalibration algorithm will take more time and this scales linearly with the number of autocalibration datapoints. The parameter estimation algorithms for noise and signal level are of lower complexity and their influence on the computation time is negligible.

## Conclusions

The goal of this paper was twofold: to propose different techniques for automation of MRI reconstruction and to develop a novel, improved autocalibration formulation for pMRI reconstruction. These goals were achieved in a single reconstruction algorithm. Firstly, the proposed algorithm was shown to benefit from the large degree of automation. An example is the the novel k-space noise level estimation results in a noise-robust reconstruction that outperforms noise-oblivious reconstruction techniques both qualitatively and quantitatively. Another example is that this is the first method (to the authors knowledge) for automatic detection of an autocalibration region and compatibility with arbitrarily sampled non-Cartesian trajectories. Automation not only facilitates in reducing the time and manpower needed to achieve a good reconstruction for a given dataset, but it also enables in automatically providing a best effort reconstruction for a given dataset without being biased by the taste of a specific user. This conclusion holds even for datasets that, to the knowledge of the author, no other algorithms succeed in providing a reconstruction for. This is because of the second novelty in this paper: a novel framework for autocalibrating pMRI, which is shown to be more robust against noise, due to regularization, more versatile, in the sense of k-space trajectory requirements and more accurate, in the sense of the underlying model, than other pMRI techniques. We demonstrated the algorithm in several examples, including an example where there is insufficiently sampled calibration data for proper autocalibrated pMRI. The code for the proposed algorithm is available upon request with the author.
